# Prefrontal TDCS does not improve working memory performance in individuals with chronic alcohol and tobacco use

**DOI:** 10.1016/j.ibneur.2026.01.011

**Published:** 2026-01-21

**Authors:** Franziska Göttgens, Ute Habel, Paul Wallheinke, Julie A. Blendy, Carmen Weidler

**Affiliations:** a Department of Psychiatry, Psychotherapy and Psychosomatics, Faculty of Medicine, RWTH Aachen, Aachen, Westphalia, North Rhine 52074, Germany; b Research Center Jülich, Institute of Neuroscience and Medicine, JARA-Institute Brain Structure Function Relationship (INM 10), Jülich, Westphalia, North Rhine 52428, Germany; c Department of Child and Youth Psychiatry, Psychotherapy and Psychosomatics, Agaplesion Diakonieklinikum, Rotenburg, Lower Saxony 27356, Germany; d Department of Systems Pharmacology and Translational Therapeutics, Perelman School of Medicine, University of Pennsylvania, PA 19104, USA

**Keywords:** Working memory, Prefrontal cortex, Transcranial direct current stimulation, Alcohol dependence, Nicotine

## Abstract

Working memory (WM) deficits are common in psychiatric disorders that are associated with decreased prefrontal cortex activity. As WM is essential for cognitive functions, deficits interfere with daily life and treatment. The lateralization of WM components remains unclear, but stimuli matching, often assessed using the n-back task, has been associated with right-hemispheric dominance. This study examines whether anodal transcranial direct current stimulation (tDCS) targeting the right dorsolateral prefrontal cortex (DLPFC) could enhance WM performance in alcohol-dependent patients (AD), tobacco users (TU) and healthy controls (HC). In a double-blind, sham-controlled study, tDCS was applied to upregulate right DLPFC activity. A total of 46 participants received anodal tDCS with a current intensity of 1.5 mA for 20 min or sham stimulation. In addition to the 7x5cm anode, a large reference electrode (10x10cm) was situated over the contralateral supraorbital area. While being stimulated, participants performed the n-back task as a measure of WM performance. Results revealed no significant differences in WM performance between active and sham stimulated participants, nor between groups, and no significant interaction between stimulation condition and group. Bayesian analysis supported the null effects. These findings do not provide evidence that single-session right DLPFC stimulation reliably enhances working memory across all stimulus types. The outcomes may have been influenced by task–stimulation mismatch, sample heterogeneity, small sample size, and stimulation parameters, which could limit the ability to detect subtle tDCS effects in both clinical and healthy populations.

## Highlights

• WM deficits in psychiatric disorders impair PFC function and hinder treatment.
• Stimulus-matching in n-back tasks is predominantly mediated by the right DLPFC.
• Chronic nicotine consumption modulates tDCS effects on cognition and working memory.
• Right DLPFC tDCS fails to enhance WM in alcohol-dependent patients and smokers.
• Right DLPFC may not be an optimal tDCS target for clinical WM enhancement.

## 1 Introduction

Working memory (WM) plays a crucial role in everyday life due to its significant impact on cognitive processes such as attention, language and memory (Assecondi et al., 2021) and is defined as a system enabling temporary storage and online manipulation of transitory information (Brunoni and Vanderhasselt, 2014, Andrews et al., 2011, Trumbo et al., 2016). WM has been proposed to consist of two short-term stores, one for verbal information and one for spatial information, and an executive component that functions as a control system operating on these short-term stores (Bunge, 2000, Collette and van der Linden, 2002). This structure allows individuals to keep information in mind while manipulating it to ensure complex thought and action (Brunoni and Vanderhasselt, 2014, Hoy et al., 2013, Nissim et al., 2019). These processes are essential for tasks such as learning, reasoning (Trumbo et al., 2016), problem solving or strategic planning (Nissim et al., 2019). The prefrontal cortex is especially involved in WM (Christodoulou et al., 2001), with increased brain activation predominantly observed in the left dorsolateral prefrontal cortex (DLPFC) (Baumert et al., 2020). Though, the issue of hemispheric lateralization is not ultimately resolved and could depend on specific WM sub-processes, the nature of the stimuli or encoding strategies employed (Trumbo et al., 2016, Talsma et al., 2001). For example, verbal WM tasks and verbal encoding strategies are supposed to be lateralized to the left hemisphere, visuospatial WM tasks to the right (Trumbo et al., 2016, Talsma et al., 2001). Especially WM tasks that require differentiation between matching and non-matching stimuli, commonly measured by n-back tasks, were found to be lateralized to the right hemisphere (Crego et al., 2010). Despite evidence of right-hemisphere involvement, most studies have focused on the left DLPFC (Trumbo et al., 2016) when investigating WM and its potential enhancement through noninvasive brain stimulation as a treatment option, leaving the role of the right DLPFC less explored (Živanović et al., 2021).

WM impairments are prevalent across various psychiatric disorders, such as major depression, bipolar disorder or attention deficit hyperactivity disorder (Millan et al., 2012), and are thought to interfere with effective treatment (Brunoni and Vanderhasselt, 2014). Treatment approaches, particularly psychotherapy, rely heavily on neurocognitive abilities such as memory, attentional capacity and problem solving to achieve cognitive restructuring and behavioral modifications. As such, efficient WM performance is crucial to ensure effective treatment implementation and adherence (Scott et al., 2017). Among psychiatric disorders, alcohol dependence (AD) is one of the conditions most strongly associated with WM impairments (Nowakowska et al., 2007). Chronic alcohol consumption can exert detrimental effects on WM performance (Park et al., 2011) by disrupting executive functions through interference with the prefrontal cortex. This disruption occurs via altered neurotransmitter systems, resulting in reduced neural activation and subsequent deficits in executive functions (Gutwinski et al., 2018). Furthermore, volume reductions in the prefrontal cortex (PFC) and the hippocampus have been specifically linked to deficits in WM in regard to alcohol use (Crego et al., 2010). Studying neural substrates of WM in AD, Kose et al (Kose et al., 2015). found increased activity in the dorsal anterior cingulate cortex and the insula, alongside decreased activity in the caudate and across the frontal cortex. Additionally, a study on intoxicated individuals revealed a decrease in prefrontal cortex and caudate activity in comparison to sober participants as well as an increase in activity within the hippocampus (Denson et al., 2018, Giancola, 2004). Collectively, these findings underscore the association between alcohol dependence and decreased PFC activity, particularly in relation to deficits in executive functions such as WM (Denson et al., 2018, Giancola, 2004). Functional WM may be critical for sustained abstinence and treatment success, as patients need to continuously adapt their learned behavioral patterns and exert control over their addictive behavior to achieve drug abstinence and treatment adherence (Giancola, 2007, Khemiri et al., 2019). Consequently, interventions aimed at enhancing WM and thereby facilitating the treatment process have gained increasing interest and are becoming more established in clinical practice (Assecondi et al., 2021).

Transcranial direct current stimulation (tDCS) is a noninvasive brain stimulation method that might enhance WM performance and could therefore be a valuable tool for treatment of WM deficits when added to cognitive training (Mancuso et al., 2016). TDCS is a safe and painless stimulation of brain tissue including the possibility of generating an effective sham condition (Andrews et al., 2011, Nissim et al., 2019, Nitsche et al., 2008). A weak electric current flow from the anode to the cathode is generated via electrodes placed on the scalp with the goal to modulate cortical excitability. For anodal stimulation, the cathode is a negatively charged electrode and used as a reference and the anode a positively charged electrode placed above the region of interest. The resulting low-intensity electric field leads to a depolarization of the membrane potential, influencing the cortical excitability without generating action potentials (Brunoni and Vanderhasselt, 2014, Nitsche et al., 2008). As such, tDCS could improve cognitive functions through the targeting of underlying, intrinsic neurophysiological processes (Hoy et al., 2013).Most tDCS studies targeting WM have focused on the left dorsolateral prefrontal cortex (DLPFC), based on its role in verbal working memory and encoding. While some individual studies reported improved accuracy in the n-back task following left DLPFC stimulation (Fregni et al., 2005), and others found enhanced spatial WM after anodal stimulation of the right DLPFC (Trumbo et al., 2016), the broader literature reveals considerable heterogeneity. Other studies observed reduced reaction times without accuracy changes after stimulation of the left, right, or bilateral DLPFC in both healthy and clinical populations (Dedoncker et al., 2016).

These inconsistent findings are reflected in meta-analyses. For example, Brunoni and Vanderhasselt (Brunoni and Vanderhasselt, 2014) concluded that tDCS can produce small to moderate positive effects on WM, particularly when targeting the left DLPFC, but effect sizes vary substantially depending on task type, stimulation parameters, and participant characteristics. Similarly, Hill et al (Hill et al., 2016). found moderate effects of anodal tDCS on WM accuracy, especially for online stimulation and participants with lower baseline performance. Importantly, Dedoncker et al (Dedoncker et al., 2016). demonstrated that stimulation site laterality (left vs. right DLPFC) does not consistently predict efficacy, suggesting that task demands and individual traits may modulate the effects of tDCS. A key factor may be the type of cognitive operation targeted. While verbal encoding typically recruits the left hemisphere, the process of stimulus matching—as required in many WM tasks including the n-back—is often linked to the right DLPFC. Importantly, stimulus matching is not exclusive to the n-back task but is a core process in paradigms such as delayed match-to-sample, Sternberg, flanker, and go/no-go tasks. Neuroimaging studies have consistently implicated the right DLPFC in such operations, especially when cognitive control, interference resolution, or decision-making are required (MacDonald et al., 2000, Garavan et al., 2002). For example, Garavan et al. (1999) found right-lateralized activation during sequential response inhibition, while MacDonald et al. (2000) linked the right DLPFC to conflict monitoring and task rule updating. These processes are central to WM performance under high demand. Complementing this evidence, several studies have shown that anodal tDCS over the right DLPFC can enhance performance in tasks requiring stimulus discrimination and cognitive control (Jacobson et al., 2011). Jacobson et al. (2011), for instance, reported improved accuracy and faster reaction times in a go/no-go task following right DLPFC stimulation, suggesting enhanced response monitoring and inhibition. Given these inconsistencies, it is crucial to identify the boundary conditions under which tDCS may effectively enhance WM. One factor that remains underexplored is the interaction between the stimulation site and the type of cognitive process engaged. While verbal encoding typically engages left-hemispheric regions, the n-back task involves not only encoding but also continuous updating and stimulus matching—executive processes that are supported by bilateral prefrontal activation. Neuroimaging studies have shown that verbal n-back tasks elicit activation in both hemispheres, with a stronger left-lateralized response for verbal material, but also consistent involvement of the right DLPFC, particularly under higher working memory load (Owen et al., 2005, Braver et al., 1997).

Based on this, we targeted the right DLPFC to investigate whether stimulation of executive control regions involved in stimulus matching could enhance WM performance. This design deliberately tests the boundary of stimulation-task congruence, as the verbal nature of the stimuli might favor left-hemispheric involvement. However, by stimulating the right DLPFC, we aimed to enhance task components—such as updating, matching, and monitoring—that are more strongly associated with right-lateralized prefrontal function, particularly in cognitively impaired populations.

Given that baseline cognitive deficits may increase responsiveness to neuromodulatory interventions, we included not only healthy controls (HC) but also alcohol-dependent (AD) patients and tobacco users (TU), which are two groups known to exhibit WM impairments. Previous studies on tDCS in these populations have primarily focused on craving reduction or abstinence, with limited research addressing cognitive outcomes such as WM performance. Moreover, chronic nicotine use has been linked to impaired cognitive control and slower response times, making TU a particularly relevant group for examining the cognitive effects of tDCS (Hill et al., 2016, Martin et al., 2014). Studies investigating tDCS in AD or tobacco users have focused on the reduction of craving and the achievement of abstinence, with limited research on the effects of tDCS on WM performance (Klauss et al., 2014, Vitor de Souza Brangioni et al., 2018, Wietschorke et al., 2016). Chronic nicotine consumption has been linked to impaired cognitive control, as exhibited by slower reaction times (Verveer et al., 2020), making tobacco users a particularly suitable target group for investigating the potential cognitive benefits of tDCS.

In a double-blind, sham-controlled study, alcohol dependent patients, chronic tobacco users, and healthy controls performed a WM task during tDCS application targeting the right DLPFC. The data set analyzed in the current study is part of a larger study exploring tDCS effects on aggression and impulsivity (Weidler et al., 2022). A large cathodal electrode was used as the reference electrode to minimize its effect on underlying brain tissue and placed above the left supraorbital area. Based on the right lateralization of subprocesses of WM such as stimuli matching (Crego et al., 2010), we expect differences in reaction time and accuracy between anodal and sham stimulation, as well as between the three participant groups. Specifically, we expect better WM performance indicated by faster reaction times and higher accuracy in the stimulation group when compared to the sham condition. This effect is likely to be more pronounced within the AD and TU group in comparison to the healthy controls. Furthermore, we expected AD patients and tobacco users to exhibit increased reaction times and lower accuracy compared to healthy individuals. Across all groups, we expect differences in WM performance between the n-back conditions, with performance declining as WM load increases. By investigating cognitive task demands and stimulation laterality, our study contributes to a more nuanced understanding of the mechanisms and potential applications of prefrontal tDCS in clinical and non-clinical populations.

## 2 Materials and methods

### 2.1 Participants

In total, 46 male, right-handed participants aged between 18 and 60 years with a mean age of 42.28 years (SD = 10.994) took part in the study. The required sample size was determined based on an a priori power analysis conducted in G*Power (version 3.1.9.7 for Windows, Heinrich-Heine-Universität Düsseldorf, Düsseldorf, Germany) for a mixed ANOVA with a 3 × 2 between-subjects design (Group: AD, TU, HC × Stimulation: active vs. sham; resulting in six between-subject cells) and three repeated measurements of the primary behavioral endpoints (n-back accuracy and reaction time across the 1-, 2-, and 3-back conditions). Although the final analysis included covariates (ANCOVA), the a priori power calculation was based on the ANOVA structure because G*Power does not support mixed-design ANCOVA; this yields a conservative and widely accepted estimate of required sample size. The analysis assumed a medium effect size (f = 0.25), an α error probability of.05, desired power of.80, a correlation of.50 among repeated measures, and a nonsphericity correction of 1. This resulted in an estimated required total sample size of N = 54 (critical F = 1.93, numerator df = 10, denominator df = 96, noncentrality parameter = 20.25, actual power =.86). Although the final recruited sample was slightly smaller (N = 46), the total sample was reasonably close to the planned size. Prior to the experiment, the three experimental groups, alcohol dependent patients (AD, n = 18), tobacco users (TU, n = 15) and healthy controls (HC, n = 13) gave their written informed consent and received compensatory payment after participation. Participants with a tobacco use of at least 10 cigarettes per day were eligible for the TU group. AD in-patients were recruited from the psychiatry ward of the University Hospital RWTH Aachen and out-patients were recruited using public advertising. The guidelines for medical research involving human subjects according to the latest version of the Code of Ethics of the World Medical Association (Declaration of Helsinki) were followed. All participants underwent screening using the Structured Clinical Interview for DSM-IV Axis I disorders (SCID I) (Wittchen et al., 1997). Inclusion criteria for AD patients required a primary diagnosis of alcohol dependence according to the International Statistical Classification of Diseases and Related Health Problems (ICD 10) (mean time passed since initial diagnosis = 16 years). Comorbidities with other psychiatric disorders were not considered exclusionary. Individuals included in the AD group had comorbid depression (*n* = 6), posttraumatic stress disorder (n = 2), social anxiety (n = 2), specific phobia (n = 1), dysthymia (n = 1) and panic disorder (n = 1). Nine AD patients reported to also consume substances other than alcohol. Participants indicated to use medications including atypical antidepressants (n = 3), benzodiazepines (n = 2), methadone (n = 2), antipsychotic medication (n = 1), selective serotonin reuptake inhibitors (n = 1) and anticonvulsants (n = 1). Healthy control participants were non-smokers and matched to the patient group according to age and years of education. HC and TU groups were screened for their alcohol consumption using the Alcohol Use Disorder Identification Test (Babor et al., 2001), and only individuals scoring below 8 (where 8 indicated suspected alcohol abuse) were included. Current neurological or psychiatric disorders excluded individuals in the HC and TU groups, while the standard exclusion criteria based on contraindications for magnetic resonance imaging MRI (e.g., metal implants) were applied to all groups.

### 2.2 Procedure

This study was conducted as part of a broader research project (Weidler et al., 2022) at RWTH Aachen University Hospital, which sought to explore the impact of prefrontal tDCS on impulsive and aggressive behavior in individuals diagnosed with alcohol use disorder. This paper focuses on the data derived from the n-back task performed during the tDCS session. The study received approval from the Ethics Committee of the Medical Faculty of the RWTH Aachen University. Upon arrival, participants were informed about the study’s objectives and relevant procedures. Moreover, they were not informed whether they were included in the active or sham stimulation condition. The investigators were also blinded to conditions. After completion of questionnaires and neuropsychological tests, participants engaged in two paradigms. Subsequently, there was a brief break lasting 5–10 min, during which participants in the AD group who consumed tobacco (N = 15) and the TU were allowed to smoke to prevent craving effects. Following the break, the n-back task was conducted during the tDCS application, and the tasks were repeated. Participants were debriefed and compensated for their participation.

### 2.3 N-back task

**Fig. 1:**
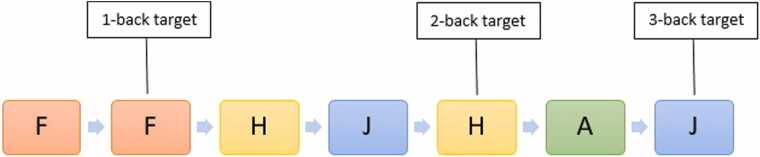
Depiction of the n-back task. Stimuli were presented for 1432 ms each leading to a total duration of 16 min. The task was divided into the 1-back, 2-back and 3-back condition, which were performed separately.

During the stimulation, the n-back task was employed to assess WM performance. Participants were seated in front of a computer where they were presented with a sequence of stimuli (alphabet letters, except for X) one at a time. Their task was to compare the current stimulus to one presented n-items earlier in the sequence. For the 2-back condition, a stimulus was considered a target if it matched the one presented two items prior. For example, in a letter sequence like “F-G-H-J…” the correct response would be “match” if the 5th letter in the sequence was “H” since it matched the one presented two positions earlier; otherwise the response would be “no match”. In total, participants completed 36 blocks. Each condition (1, 2 and 3-back) was represented by 12 blocks, each containing 19 trials. The order of the n-back conditions was randomized. The duration between the presented stimuli was set at 1432 ms, resulting in a 16-minute duration for the entire task. We evaluated WM performance by calculating both accuracy and mean reaction time for correct responses in each condition for every participant. Accuracy was calculated by dividing the number of correct responses by the total possible responses for each condition. An illustration of the task is shown in Fig. 1.

### 2.4 TDCS

**Fig. 2:**
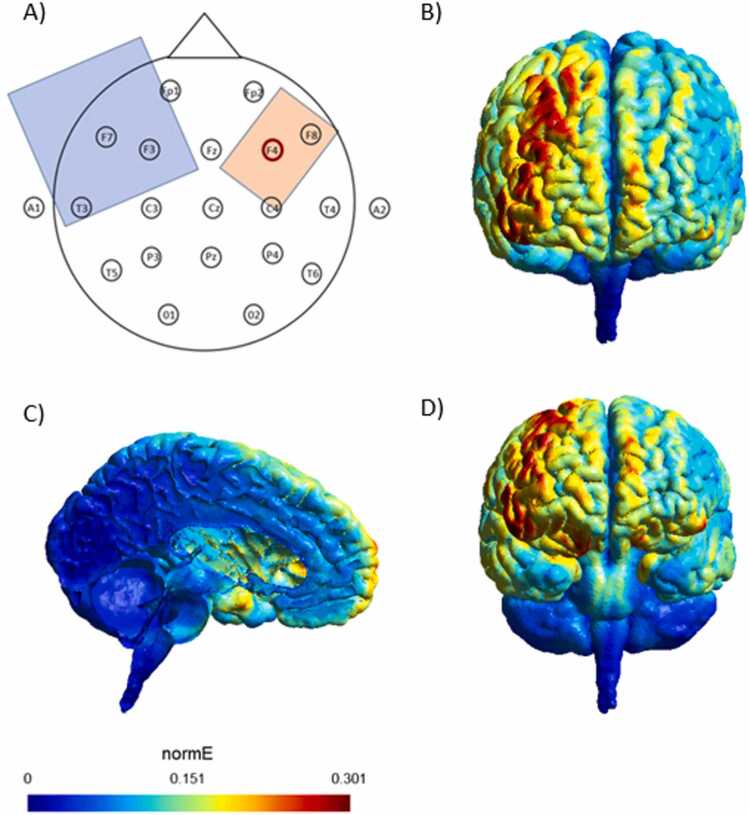
A) Schematic illustration of the electrode montage, where the cathodal electrode was positioned on the supraorbital area on the left hemisphere and the anodal electrode on the F4 position of the EEG 10–20 cap, which corresponds to the right DLPFC. B) Simulation of the resulting electrical fields with SIMNIBS 3.2, showing a current density of 0.06 mA/cm² (0.6 A/m²) under the anode and 0.015 mA/cm² (0.15 A/m²) under the cathode. C) Frontal view of the left hemisphere illustrating the simulated electrical field under the cathodal electrode. D) Posterior-tilted view highlighting the orbitofrontal cortex (OFC) region. The simulations show that while the maximal electric field is concentrated over the right DLPFC, some current reaches the OFC under the left reference electrode, suggesting moderate stimulation effects.

The stimulation started ahead of the n-back task, utilizing a battery-driven, constant current stimulator (NeuroConn, Erlangen, Germany). TDCS was applied to the scalp through two rubber electrodes placed in saline-soaked sponges using NaCl as conductive medium. The anode (5 ×7 cm²) was placed over the F4 location of the EEG 10/20 system to stimulate the right DLPFC, while the cathode, serving as a reference electrode (10 ×10 cm²), was placed over the left supraorbital area, maintaining a minimum distance of 7 cm from the anode (see Fig. 2). In the active tDCS group, current was delivered with an intensity of 1.5 mA for 20 min. Current intensity was gradually ramped-up over a period of 20 s at the beginning and faded out gradually for 20 s at the end of the stimulation period. The n-back task began 4 min after the stimulation started and continued for 16 min. Stimulation was applied online, meaning that participants performed the cognitive task during the tDCS session. This approach was chosen to engage the participants in active cognitive processing during stimulation, which has been shown to enhance task-related neural modulation compared with offline protocols (Nikolin et al., 2018). Post-stimulation effects were not assessed in this study, as the primary focus on aftereffects was related to aggression, measured separately. The same parameters were applied to the sham tDCS group, except that stimulation was terminated after 20 s. Consequently, all participants experienced the initial itching sensations and were therefore unlikely to recognize whether they were actively stimulated during the remaining session or not. TDCS was performed in a double-blind manner, ensuring that both the experimenter and the participants remained unaware of the type of stimulation being administered. Double-blinding was implemented using device codes generated by the principal investigator; the experimenters conducting the sessions had access to these codes but were unaware of whether participants received active or sham stimulation. A Chi-square test of guessed condition confirmed that participants were unable to reliably distinguish between active and sham stimulation. Participants completed a standardized post-stimulation questionnaire assessing common tDCS-related sensations.

### 2.5 Statistical analysis

Two repeated-measures ANCOVAs were used to compare the influence of tDCS versus sham stimulation on WM performance in three groups. The mean reaction time and accuracy were treated as dependent variables, with the type of WM task (1-back, 2-back, 3-back) as the within-subjects factor, and tDCS and the experimental group (AD, HC and TU) as the between-subjects factors. Medication and comorbidities were included as covariates. Effect sizes were reported using partial eta-squared. Posthoc tests were adjusted using the Bonferroni correction, the Huynh-Feldt correction was applied when sphericity was violated. Additionally, a Chi-square test was performed to assess the effectiveness of tDCS blinding. Additionally, an examination utilizing Bayesian analysis was carried out to enhance insights into outcome probabilities. Bayesian ANOVAs were implemented to assess the strength of evidence supporting the alternative hypothesis (H_1_) as opposed to the null hypothesis (H_0_). By employing Bayes factors, we quantified the extent to which the data supports H_1_ over H_0_ (BF_10_). A BF_10_ exceeding 3 indicates support for H_1_, while a BF_10_ between 1/3 and 3 suggests a lack of sensitivity in differentiating between H_0_ and H_1_. Lastly, a BF_10_ below 0.33 indicates substantial support for the null hypothesis (Perry, 2016). Statistical analyses were performed with SPSS (IBM SPSS Statistics 52.0; Ehningen; Germany), while the figure was created using R (R Core Team, 2014). Sample characteristics are presented in Table 1.

**Table 1:** Descriptives (Mean ± SD). SD = Standard deviation; HC = healthy controls; AD = alcohol-dependent patients; TU = tobacco users; tDCS = transcranial direct current stimulation.

	HC	AD	TU	Total	
N	13	18	15	46	
Age	41.69 ± 10.62	42.33 ± 10.77	42.73 ± 12.27	42.28 ± 10.99	
Accuracy in %					
1-back sham	91.48 ± 6.2	91.72 ± 11.28	92.98 ± 5.04	92.07 ± 7.9	
1-back tDCS	93.64 ± 3.49	90.74 ± 4.9	91.29 ± 10.16	91.71 ± 6.56	
2-back sham	81.64 ± 10.45	86.40 ± 10.58	85.64 ± 8.42	84.76 ± 9.66	
2-back tDCS	85.45 ± 7.96	79.73 ± 7.73	84.02 ± 9.38	82.66 ± 8.34	
3-back sham	78.45 ± 8.71	78.07 ± 7.5	77.52 ± 8.97	77.9 ± 7.9	
3-back tDCS	76.61 ± 6.4	72.2 ± 6.71	79.45 ± 10.59	75.75 ± 8.31	
Reaction time in ms					
1-back sham	486.33 ± 84.25	461.40 ± 46.59	441.8 ± 47.6	462.17 ± 60.17	
1-back tDCS	471.07 ± 46.01	438.5 ± 77.59	491.42 ± 114.99	464.26 ± 84.36	
2-back sham	523.68 ± 57.71	506.27 ± 57.46	479.12 ± 43.78	502.29 ± 54.16	
2-back tDCS	518.96 ± 29.18	507.25 ± 95.80	486.96 ± 75.2	503.99 ± 74.03	
3-back sham	472.19 ± 27.37	512.59 ± 80.91	458.45 ± 52.25	482.76 ± 62.38	
3-back tDCS	526.85 ± 65.76	487.83 ± 118.76	496.27 ± 77.29	501.16 ± 91.55	

## 3 Results

### 3.1 Accuracy

**Fig. 3:**
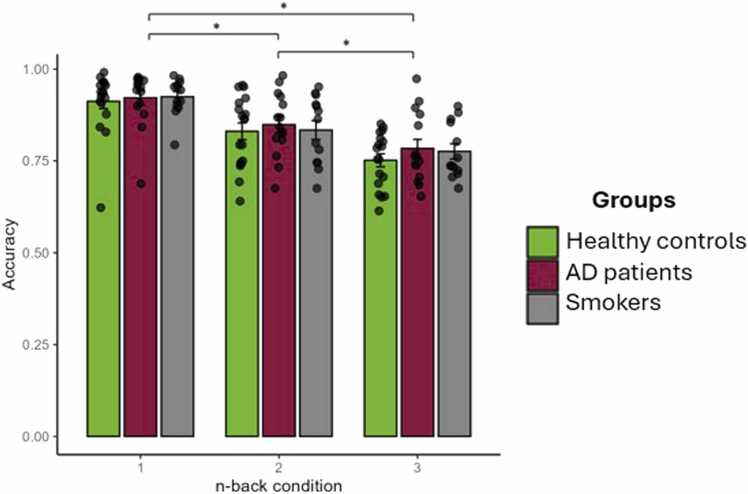
Results of the repeated-measures ANCOVA regarding the mean accuracy per group (HC, AD, TU) and n-back condition (1-back, 2-back and 3-back). Error bars represent the standard error. The mean difference is significant at the.05 level. The groups did not differ from each other within the n-back conditions. The n-back conditions among each other reached significance (*p* < .001).

As a measure of working memory performance, accuracy per condition was examined using a repeated-measures ANCOVA with medication and psychiatric comorbidities included as covariates to adjust the effects of tDCS and group. This resulted in a significant main effect of n-back condition (*F*(2, 76) = 83.946, *p* < .001, η²ₚ = .688, CI [.561,.758]), while no significant interaction effects could be found between the n-back condition and tDCS (*F*(2, 76) = .405, *p* = .668, η²ₚ = .011, CI [0,.074]), n-back condition and group (*F*(4, 76) = .418, *p* = .795, η²ₚ = .022, CI [0,.065]) or between all three of them (*F*(4, 76) = 2.130, *p* = .085, η²ₚ = .101, CI [0,.201]; see Fig. 3). Reported effects of tDCS and group are therefore adjusted for medication and comorbidities which had no significant effects on either stimulation or WM. Post hoc pairwise comparisons using the Bonferroni adjustment indicated significant differences between 1-back (*M* =.923, *SD* =.011) and 2-back conditions (*M* =.840, *SD* =.014); *p* < .001, 1-back and 3-back conditions (*M* =.772, *SD* =.012); *p* < .001 as well as 2-back and 3-back conditions (*p* < .001). A Bayesian ANOVA found substantial support for the null hypothesis regarding 1back (BF_10_ =.023), 2back (BF_10_ =.024) and 3back conditions (BF_10_ =.040). Hence, this supports the conclusion that there were no meaningful differences in accuracy among the three groups, regardless of whether they underwent active or sham stimulation across all n-back conditions.

### 3.2 Reaction time

The repeated-measures ANCOVA assessing the influence of stimulation (tDCS vs. sham) and group (HC vs. AD vs. TU) on mean reaction time per condition included medication and psychiatric comorbidities as covariates to adjust the effects of tDCS and group. This analysis revealed no significant main effect of n-back condition (*F*(1.846, 70.153) = 2.580, *p* = .087, η²ₚ = .064, CI [0,.181]). Mauchly’s test indicated a violation of sphericity, and Huynh-Feldt–corrected degrees of freedom were used for all within-subjects effects. No significant interactions between n-back condition and tDCS (*F*(1.846, 70.153) = .847, *p* = .425, η²ₚ = .022, CI [0,.109]), n-back condition and experimental group (*F*(3.692, 70.153) = .446, *p* = .760, η²ₚ = .023, CI [0,.075]) or between n-back condition, tDCS and group (*F*(3.692, 70.153) = 1.626, *p* = .181, η²ₚ = .079, CI [0,.177]) were found. Reported effects of tDCS and group are therefore adjusted for medication and comorbidities which had no significant effects on either stimulation or WM. A Bayesian ANOVA found substantial support for the null hypothesis regarding 1back (BF_10_ =.037), 2back (BF_10_ =.075) and 3back conditions (BF_10_ =.031). Consequently, there is substantial support for the conclusion that there were no substantial differences in reaction time among the three groups, whether subjected to active or sham stimulation across all n-back conditions.

### 3.3 Blinding

To assess whether the blinding of stimulation conditions (active vs. sham) was successful, participants were asked after completion of the task what condition they received. A Chi-square test was conducted indicating no interaction between the stimulation condition and the condition they thought they received, X² (1, *N* = 46) = 2.113, *p* = .146.

In addition, participants reported the intensity of stimulation-related sensations, including warmth, numbness, pain, itching, burning, and tingling (see Table 2). Sensations were generally low across both sham and tDCS conditions, with slightly higher reports of tingling and itching during tDCS. Overall, these results indicate that stimulation was well tolerated and that the sham protocol effectively mimicked the sensory experience of tDCS, supporting the integrity of the blinding.

**Table 2:** Mean ± SD ratings of stimulation-related sensations reported by participants during Sham and tDCS conditions.

Sensation	Sham	tDCS
Warmth	1.64 ±.90	1.62 ±.67
Numbness	1.00 ±.00	1.19 ±.40
Pain	1.09 ±.29	1.28 ±.56
Itching	1.32 ±.72	1.76 ± 1.13
Burning	1.33 ±.68	1.52 ±.75
Tingling	1.77 ±.68	2.14 ± 1.01

## 4 Discussion

The present study investigated whether anodal tDCS over the right DLPFC could enhance WM performance in an n-back task by targeting a region typically associated with stimulus matching, a process considered right-lateralized, we aimed to probe the boundary conditions of tDCS efficacy. Our decision to use verbal stimuli, despite their left-hemispheric processing dominance, intentionally created a task-stimulation incongruence to test whether right DLPFC stimulation could nontheless enhance performance. By including alcohol-dependent (AD) patients, chronic tobacco users (TU), and healthy controls (HC), we aimed to assess the effects of stimulation across groups with differing baseline cognitive profiles. As expected, WM performance deteriorated with increasing workload for all three experimental groups, supporting the increased WM load across the three n-back conditions. However, contrary to our hypotheses, AD and TU participants showed no significant deficits in accuracy or reaction time compared to HCs. Additionally, there was no significant difference between active and sham stimulation across all groups, suggesting that a single session of anodal tDCS over the right DLPFC did not produce measurable enhancement of WM performance in this mixed clinical sample.

In contrast to our hypothesis, the findings indicated that AD, TU, and healthy control groups demonstrated comparable accuracy and reaction times across all three n-back conditions. The absence of group differences contrasts with existing research, which frequently highlights working memory deficits in individuals with AD and TU (Crego et al., 2010). Several factors may explain this. One-third of the AD group had been abstinent for different lengths of time. Abstinence is known to facilitate neurogenesis and cognitive recovery (Gazdzinski et al., 2005), potentially leading to the reduction of previously enlarged sulci and ventricles, the restoration of myelination and axonal wiring, and an increase of total brain volume and the grey matter in the PFC (Fein et al., 2006). Fein et al (Grundey et al., 2015). found similar cognitive functioning in long-term abstinent patients compared to a control group matched in age and gender, with no significant differences in WM between the groups. This may help to explain the absence of significant differences between our AD sample and the control group, suggesting that abstinence may have contributed to the restoration of WM deficits. Additionally, nicotine use among the AD and TU group may have influenced WM. Several studies have shown that nicotine can enhance cognitive functions such as attention, memory and information processing (Ettinger et al., 2009). Acute nicotine administration has been associated with improved WM in smokers, (Wylie et al., 2012, Ernst et al., 2001), potentially elevating performance levels in smoking AD and TU participants to those of the control group. However, the effects of nicotine are complex; while it may improve WM performance in smokers during withdrawal, it can impair WM in non-smokers (Grundey et al., 2015, Wylie et al., 2012). The absence of WM deficits in the AD and TU groups may partly reflect the immediate effects of nicotine in participants who smoked before testing. Taken together, these findings suggest that abstinence and nicotine use may have mitigated WM deficits in the AD and TU groups, potentially equalizing their performance with that of the control group. The combined effects of abstinence and nicotine use may have masked expected group differences.

While factors such as abstinence, nicotine use, and other neurocognitive influences may explain the lack of group differences in our study, the lateralization of WM sub-processes could also play a role, particularly in regard to the lack of significant tDCS effects.

WM relies on a network of interconnected brain regions, with specific sub-processes being lateralized across hemispheres. This lateralization could influence the efficacy of tDCS, particularly when the stimulation is applied to only one hemisphere, potentially limiting its impact on overall WM performance (Crego et al., 2010). Specifically, our investigation focused on stimulating the right DLPFC, a region that has been less explored in the context of WM enhancement compared to the left DLPFC (Mancuso et al., 2016). While activation of the prefrontal cortex is generally associated with WM across its various components (Christodoulou et al., 2001), hemispheric lateralization may differ depending on the specific WM processes engaged. Specifically, stimulus matching is lateralized to the right hemisphere (Crego et al., 2010). Talsma et al (Talsma et al., 2001). reported that the identification of a test target is predominantly left-lateralized. Both stimulus matching and target identification are integral to the n-back task, potentially recruiting both hemispheres. Additionally, the left hemisphere is typically linked to processing language-based stimuli, while the right hemisphere is more involved in visuospatial stimuli (Müller et al., 2022). Given that our n-back task utilized letters as targets and thereby emphasizing verbal WM, this could account for the lack of improvement in WM performance, given that the right DLPFC was stimulated. A meta-analysis by Müller et al (Smirni et al., 2015). reviewed several studies on high-definition tDCS targeting the DLPFC and found an effect of left DLPFC stimulation. Other studies have also reported enhanced WM performance following tDCS targeting the left DLPFC in healthy as well as clinical samples, such as patients with schizophrenia or Parkinson’s disease (Andrews et al., 2011). These findings suggest that stimulation of the left DLPFC might have been more effective for enhancing verbal WM performance (Hoy et al., 2013). Our non-significant findings align with some previous studies reporting limited or variable effects of right DLPFC stimulation on WM, highlighting the importance of task-stimulation alignment and other boundary conditions (Tremblay et al., 2014). Nevertheless, it is important to note that several studies have reported enhancements in cognitive functions, such as WM performance (Meiron and Lavidor, 2013), following anodal stimulation of the right DLPFC. For instance, a study examining the online effects of anodal stimulation of the right DLPFC found a beneficial effect for high-load verbal WM maintenance but only in female participants (Wang et al., 2018). Similarly, Li et al. (2017) observed significant improvements of visual WM performance following stimulation of the right DLPFC, while Wang et al. (2018) observed a suppressive effect on WM maintenance, as evidenced by a lower hit rate (Lally et al., 2013). However, a recent meta-analysis identified minimal effects of right lateralized brain stimulation on WM though they reviewed studies using transcranial alternating current stimulation (Mancuso et al., 2016). These conflicting results highlight the complexities of DLPFC stimulation, emphasizing the need to refine strategies and further explore its role in enhancing WM. The lack of significant tDCS effects highlights the importance of considering task-stimulation congruence. While stimulus matching processes are often right-lateralized, the verbal nature of our stimuli may have primarily engaged left-hemispheric regions. Consequently, stimulating the right DLPFC may not have optimally targeted the neural substrates involved in task performance. Given the limitations of our study, including small sample size, sample heterogeneity, and possible task–stimulation mismatches, this interpretation should be made cautiously. Consequently, our data does not provide evidence that stimulating the right DLPFC reliably enhances performance on this verbal n-back task in this mixed clinical sample. Although some studies have reported benefits of right DLPFC stimulation under certain conditions (Wang et al., 2018, Lally et al., 2013), overall, the current evidence suggests that the alignment between stimulation site and cognitive process is critical for achieving cognitive enhancement.

In addition to hemispheric lateralization, specific tDCS parameters may have contributed to the lack of significant differences in WM accuracy and reaction time between the active and sham stimulation condition. Our study exclusively examined the immediate online effects during stimulation, precluding any conclusions about potential after-effects. Previous research has highlighted that while stimulation may not lead to enduring improvements in WM beyond the stimulation period, beneficial effects can still be observed during task performance, suggesting the presence of early learning effects (Stagg et al., 2011). Although research on the temporal dynamics of frontal tDCS effects suggests that brain activation tends to be more pronounced during the stimulation period compared to the post-stimulation phase (Nikolin et al., 2018), WM improvements are more often reliably observed after the stimulation ended than during the stimulation (Baumert et al., 2020). For example, a study by Baumert et al (Baumert et al., 2020). found enhanced WM performance only 15 min after anodal tDCS over the left DLPFC. Since our study only investigated online effects of stimulation, this may account for the lack of significant differences observed between stimulation conditions. This aligns with prior findings that suggest online effects may be less robust than those emerging after stimulation. Also, it might be reasonable to include more than a single tDCS session or to use the stimulation repeatedly in addition to cognitive training sessions (Mancuso et al., 2016, Nilsson et al., 2015). Another factor that may have contributed to the lack of observed tDCS effects is the electrode montage used in the present study. The relatively large return electrode was placed over the left supraorbital area. While a larger return electrode is often used to reduce current density and minimize unintended stimulation, it also reduces focality and does not entirely eliminate neuromodulatory effects under the return site. E-field simulations of this montage indicate that the maximal electric field was concentrated over the right DLPFC, but some current reached the OFC under the left reference electrode. Thus, although the impact on the primary regions of interest was likely limited, the left reference electrode may still have exerted moderate stimulation effects. Consequently, this montage may have inadvertently influenced left prefrontal regions involved in verbal WM processing, potentially counteracting or diffusing the effects of anodal stimulation over the right DLPFC. Future studies might benefit from employing high-definition or more focal montages to better isolate stimulation effects and clarify the role of lateralized tDCS in cognitive enhancement.

Another factor to consider is the interaction between tDCS and nicotine intake, which could have further influenced cognitive outcomes. Nicotine affects cognitive functions by modulating neuroplasticity, primarily through influencing calcium permeability and the activation of acetylcholine receptors. Similarly, tDCS modifies cortical excitability through comparable calcium-related mechanisms. However, nicotine-induced calcium influx might lead to calcium overload within neurons, disrupting the homeostasis required for tDCS-induced plasticity to occur effectively (Grundey et al., 2012, Grundey et al., 2018). This interference likely impairs the brain's ability to establish the synaptic changes required for sustained WM improvements. In our study, nicotine intake among the TU and AD participants could have counteracted the effects of tDCS, potentially explaining why no significant differences were observed, even in groups that are typically more responsive to brain stimulation. While nicotine use has been shown to transiently enhance some cognitive functions, such as attention and memory, its interaction with tDCS is not straightforward (Sacco et al., 2004, Brunelin et al., 2015). The overlapping calcium-mediated pathways may have resulted in interference, where nicotine's acute effects diminishes the slower, gradual neuroplastic changes induced by tDCS (Grundey et al., 2012, Grundey et al., 2018). Additionally, nicotine use introduces heterogeneity in how participants respond to brain stimulation. Chronic nicotine exposure can lead to long-term neuroadaptive changes, such as receptor desensitization or altered neurotransmitter release, which might further complicate the interaction with tDCS (Martin-Soelch, 2013, Wills and Kenny, 2021). Such variability could mask potential tDCS effects, especially in mixed populations where baseline neural excitability differs significantly. This interaction underscores the need to investigate how substance use influences the efficacy of neuromodulatory techniques like tDCS. Future research should prioritize understanding these mechanisms, not only by studying the acute effects of nicotine but also by examining its long-term impacts on brain stimulation outcomes. For instance, controlling for nicotine use, comparing responses between nicotine-naïve and dependent individuals, or testing different stimulation protocols may help clarify these interactions.

Finally, it is important to note that individuals with neurological or psychiatric conditions often exhibit more pronounced effects of tDCS compared to healthy participants, as their impaired neural networks may provide a greater potential for modulation (Verveer et al., 2020, Grundey et al., 2018). In contrast, healthy individuals typically have well-functioning neural networks, which may limit the observable impact of tDCS. A meta-analysis showed that stimulation effects can vary based on whether they were conducted in healthy or clinical samples, as well as the specific tDCS parameters used (Dedoncker et al., 2016). Furthermore, studies on electric field distributions found differences between healthy individuals and patients, which may contribute to these varying effects. For example, patients with neuropsychiatric conditions often exhibit altered anatomical and physiological characteristics, such as cortical atrophy, which can affect the distribution and intensity of the e-field, potentially enhancing the efficacy of tDCS in these groups (Mizutani-Tiebel et al., 2022). Based on these general findings, we expected differential tDCS effects across the AD, TU, and healthy control groups in our study. Contrary to this expectation, our study found that the AD, TU, and healthy control groups demonstrated comparable accuracy and reaction times across all three n-back conditions, suggesting that tDCS did not produce the expected differential effects between these groups. Thus, there were no significant deficits within the AD and TU groups compared to the healthy controls. However, given the small sample size, group heterogeneity, and limited power to detect subtle or group-specific effects, these null findings should be interpreted with caution. Several factors might explain this lack of significant differences between groups, including hemispheric lateralization, abstinence or nicotine use. Abstinence in the AD group could have contributed to neurogenesis, brain structure restoration, and cognitive recovery, leading to comparable WM performance with the control group (Wylie et al., 2012, Müller et al., 2022). Similarly, nicotine's cognitive-enhancing effects may have elevated WM performance in smoking participants within the AD and TU groups to levels comparable to controls (Grundey et al., 2015). Additionally, the lack of significant tDCS effects on WM performance may be linked to the lateralization of WM sub-processes. Stimulation of the right DLPFC, a region less associated with verbal WM tasks, may have limited its impact on processes predominantly mediated by the left hemisphere, such as target identification and language-based stimulus processing (Park et al., 2011, Mancuso et al., 2016). Furthermore, the expectation that clinical samples would consistently benefit from tDCS when no effects are observed in healthy samples has not been consistently supported, as evidenced by the null results in our study and other investigations (Cummine et al., 2020, Jacoby and Lavidor, 2018, Horvath et al., 2015). Such insights are essential for tailoring tDCS interventions to populations with varying substance use profiles and for identifying conditions under which tDCS may remain effective despite the presence of confounding factors like nicotine.

In conclusion, our findings highlight the complexity of hemispheric lateralization in WM, with different sub-processes appearing to be lateralized to different hemispheres. Specifically, stimulus matching seems to be right-lateralized, while target identification and verbal processing are more associated with the left hemisphere, which might explain why stimulating the right DLPFC did not yield significant results. These conflicting results underline the complexities in studying the effects of DLPFC stimulation. While our study did not find significant improvements, the presence of effects in some studies emphasizes the need for further exploration into the role of right DLPFC stimulation in WM enhancement, as well as the need to refine stimulation strategies to better target specific WM mechanisms. Additionally, the effectiveness of tDCS may depend on various parameters, including the timing protocol of stimulation and its interaction with sample characteristics and substance use. The present study refines our understanding of the conditions under which tDCS may or may not enhance WM. Future research should prioritize stimulation-task matching, explore post-stimulation effects, and consider the moderating impact of substances such as nicotine to optimize the use of tDCS for cognitive enhancement.

## 5 Limitations and future directions

The study is limited to an all-male sample excluding the potential differences in genders being affected by tDCS (Rudroff et al., 2020). Another important aspect to consider is that the participants already engaged in two cognitive tasks before the n-back task, potentially introducing effects unaccounted for. Moreover, our study has limitations related to sample characteristics. The absence of group differences may be influenced by the inclusion of smokers among AD patients (n = 15), as nicotine is known to affect cognitive functions, including working memory. Future research should explore the interactions between nicotine and tDCS and investigate underlying molecular mechanisms. Furthermore, the relatively small and uneven group sizes may have reduced the statistical power to detect subtle effects. Given that the power analysis was based on a medium effect size and that tDCS studies typically report small effects (Perry, 2016), such small effects would likely not have been detectable with the present sample size. Moreover, we only examined online tDCS effects during task performance and did not assess potential aftereffects on working memory, which have been reported on previous studies (Weidler et al., 2022); future studies should consider including follow-up testing to capture delayed tDCS benefits. Additional limitations include variations in addiction stages among AD patients. Differentiating between individuals in abstinent and acute stages of AD should be considered in future studies.

## 6 Conclusion

In conclusion, our findings do not provide evidence for reliable online enhancement of verbal n-back performance by a single session of right DLPFC tDCS in this mixed clinical sample. Several boundary conditions likely influenced the outcomes, including hemispheric lateralization of WM sub-processes, task-stimulation incongruence, sample heterogeneity, nicotine use, and varying abstinence periods among AD participants. These findings emphasize the need for further research to refine stimulation strategies, optimize task-stimulation alignment, and systematically explore the conditions under which right DLPFC tDCS may enhance WM. By targeting those brain regions that show reduced activity in AD, WM performance could be enhanced by brain stimulation over multiple sessions or in addition to cognitive training. Sample characteristics should be taken into account, especially nicotine. In conclusion, the present study offers important insights into the limits of right DLPFC stimulation for enhancing verbal working memory performance. Despite previous evidence suggesting right-hemispheric involvement in stimulus matching, our findings indicate that when verbal stimuli are used, targeting the right DLPFC may not be sufficient to improve working memory, even in clinical groups with presumed deficits. This highlights the critical role of task-stimulation congruence in neuromodulation research. Rather than viewing null effects as merely negative outcomes, our results refine the theoretical framework guiding tDCS interventions, emphasizing the need to tailor stimulation parameters carefully to task demands and target population characteristics. Future studies should continue to systematically investigate how lateralization, stimulus modality, and clinical factors interact to determine the success of neuromodulatory techniques. By clarifying these boundary conditions, we can move toward more effective and individualized applications of brain stimulation to support cognitive functions.

## CRediT authorship contribution statement

**Franziska Göttgens:** Writing – original draft, Formal analysis. **Carmen Weidler:** Writing – review & editing, Investigation, Conceptualization. **Paul Wallheinke:** Investigation, Formal analysis. **Ute Habel:** Writing – review & editing, Funding acquisition, Conceptualization. **Julie A. Blendy:** Conceptualization.

## Funding

The study was funded by the Deutsche Forschungsgemeinschaft (DFG, 10.13039/501100001659German Research Foundation – 269953372/GRK2150).

## Declaration of Competing Interest

The author(s) declare no competing interests.

## Data Availability

The dataset analyzed during the current study is not publicly available because we do not have the ethics vote for sharing the data but are available from the corresponding author on reasonable request.
